# Supplementary material for: Resolving the full spectrum of human genome variation using Linked-Reads
Source: Genome Res. 2019 Apr;29(4):635–45. doi: 10.1101/gr.234443.118 (PMC6442396; doi:10.1101/gr.234443.118)
Supplement: Supplemental Material [file supp_gr.234443.118_Supplemental_longranger-2.2.zip › longranger-2.2.tar.bz2!/lr2.2-public/lib/stan/cmdstan/stan/doc/api/html/structstan_1_1gm_1_1statement__grammar-members.html]

Stan: Member List


|  |  |
| --- | --- |
|  | Stan  2.5.0  probability, sampling & optimization |


- Main Page
- Related Pages
- Namespaces
- Classes
- Files

- Class List
- Class Index
- Class Hierarchy
- Class Members

All Classes Namespaces Files Functions Variables Typedefs Enumerations Enumerator Friends Macros Pages

- stan
- gm
- statement\_grammar

stan::gm::statement\_grammar< Iterator > Member List

This is the complete list of members for stan::gm::statement\_grammar< Iterator >, including all inherited members.

|  |  |  |
| --- | --- | --- |
| assignment\_r | stan::gm::statement\_grammar< Iterator > |  |
| dims\_r | stan::gm::statement\_grammar< Iterator > |  |
| distribution\_r | stan::gm::statement\_grammar< Iterator > |  |
| error\_msgs\_ | stan::gm::statement\_grammar< Iterator > |  |
| expression\_g | stan::gm::statement\_grammar< Iterator > |  |
| for\_statement\_r | stan::gm::statement\_grammar< Iterator > |  |
| identifier\_r | stan::gm::statement\_grammar< Iterator > |  |
| increment\_log\_prob\_statement\_r | stan::gm::statement\_grammar< Iterator > |  |
| local\_var\_decls\_r | stan::gm::statement\_grammar< Iterator > |  |
| no\_op\_statement\_r | stan::gm::statement\_grammar< Iterator > |  |
| non\_lvalue\_assign\_r | stan::gm::statement\_grammar< Iterator > |  |
| opt\_dims\_r | stan::gm::statement\_grammar< Iterator > |  |
| print\_statement\_r | stan::gm::statement\_grammar< Iterator > |  |
| printable\_r | stan::gm::statement\_grammar< Iterator > |  |
| printable\_string\_r | stan::gm::statement\_grammar< Iterator > |  |
| range\_r | stan::gm::statement\_grammar< Iterator > |  |
| reject\_statement\_r | stan::gm::statement\_grammar< Iterator > |  |
| return\_statement\_r | stan::gm::statement\_grammar< Iterator > |  |
| sample\_r | stan::gm::statement\_grammar< Iterator > |  |
| statement\_2\_g | stan::gm::statement\_grammar< Iterator > |  |
| statement\_grammar(variable\_map &var\_map, std::stringstream &error\_msgs) | stan::gm::statement\_grammar< Iterator > |  |
| statement\_r | stan::gm::statement\_grammar< Iterator > |  |
| statement\_seq\_r | stan::gm::statement\_grammar< Iterator > |  |
| truncation\_range\_r | stan::gm::statement\_grammar< Iterator > |  |
| var\_decls\_g | stan::gm::statement\_grammar< Iterator > |  |
| var\_lhs\_r | stan::gm::statement\_grammar< Iterator > |  |
| var\_map\_ | stan::gm::statement\_grammar< Iterator > |  |
| void\_return\_statement\_r | stan::gm::statement\_grammar< Iterator > |  |
| while\_statement\_r | stan::gm::statement\_grammar< Iterator > |  |


---

[ Stan Home Page ]

*© 2011–2014,
Stan Development Team.*
